# A Novel Fusion-Based Ship Detection Method from Pol-SAR Images

**DOI:** 10.3390/s151025072

**Published:** 2015-09-29

**Authors:** Wenguang Wang, Yu Ji, Xiaoxia Lin

**Affiliations:** 1 Institute of Electronic and Information Engineering, Beihang University, Beijing 100191, China; E-Mail: darlonly@163.com; 2 Lucent Technology Investment Limited, Beijing 100073, China; E-Mail: linxx@126.com

**Keywords:** Pol-SAR, ship detection, target decomposition, difference degree

## Abstract

A novel fusion-based ship detection method from polarimetric Synthetic Aperture Radar (Pol-SAR) images is proposed in this paper. After feature extraction and constant false alarm rate (CFAR) detection, the detection results of HH channel, diplane scattering by Pauli decomposition and helical factor by Barnes decomposition are fused together. The confirmed targets and potential target pixels can be obtained after the fusion process. Using the difference degree of the target, potential target pixels can be classified. The fusion-based ship detection method works accurately by utilizing three different features comprehensively. The result of applying the technique to measured Airborne Synthetic Radar (AIRSAR) data shows that the novel detection method can achieve better performance in both ship’s detection and ship’s shape preservation compared to the result of K-means clustering method and the Notch Filter method.

## 1. Introduction

Synthetic Aperture Radar (SAR) imagery has the capability of providing high resolution images for a large area under all-weather and day and night conditions. This makes SAR an important remote sensing tool to observe man-made metallic targets over sea. Therefore, ship detection based on SAR images has significantly broad applications in areas such as fisheries, water traffic and military detection for ships, due to the differences in structure and material, appear in SAR images as bright spots over a dark marine background. Although there are speckles caused by multiplicative noise and some interfering zones which also appear bright as potential targets in SAR images, polarimetric SAR (Pol-SAR) images, which contain multiple channels, still can provide more information about target characteristics compared with traditional single polarized SAR images. Hence, maritime ship detection based on Pol-SAR images is becoming an important field in the research of SAR images.

Conventional ship detection methods are based on single polarized SAR images. Constant false alarm rate detection and radon transformation are two main methods to detect ships [1,2]. For Pol-SAR images, Liu [3] and Touzi *et al.* [4] applied the polarimetric whitening filter (PWF), Optimal Polarimetric Detector (OPD) and SPAN detectors for ship detection, and demonstrated that some Pol-SAR images methods can better detect the targets than the traditional single polarized SAR method. The improvement can be interpreted from the basic fact that Pol-SAR images are able to not only provide the radar scattering cross-section (RCS), but also some polarimetric information, such as the scattering mechanism, target structure, target orientation angle, *etc*. Many features extracted from Pol-SAR images can reveal the differences between targets and background from various aspects. This also makes ship detection based on polarimetric features a research hotspot. For instance, Ringrose [5] used the Cameron decomposition method [6] to detect ships from SIR-C data, which was considered the first to apply the polarization decomposition to ship detection. They also analyzed the feasibility of employing target scattering features in ship detection, where they pointed out that the ocean was made up of cylinder scattering while ships were mainly composed of dihedral, narrow dihedral and 1/4 waves scattering. This method had certain application prospects in the low resolution image field, but Touzi [7] proved that this method had defects in practical applications. For instance, only part of the ocean pixels can be classified as cylinder scattering, while a large number of ship pixels will be put into the unknown classes. A paradigm based on the different symmetry properties of sea and man-made targets is presented in [8], which exploited the intrinsic physical features that characterize man-made metallic targets. Nunziata then implemented a CFAR approach to exploit Cosmo-Skymed (CSK) PingPong full-resolution SAR data for observation purposes, based on a model that related the time offset between co-polarized (HH/VV) bursts to the scene coherence time with and without targets [9]. In 2001, Yeremy [10] adopted Cloude decomposition in the marine environment and obtained better results for ship detection and recognition by combining it with van Zyl decomposition. Li [11] proposed an improved ship detection method based on 2D convolution between co-pol channels. Wang [12] proposed a ship detection method based on feature vectors, which included two steps of classification and recognition. Marino [13] proposed a ship detection method using the Notch Filter, in which all the signatures that possessed a polarimetric behavior different from the sea were detected. Two statistical tests for the ship detector based on the Geometrical Perturbation-Polarimetric Notch Filter were devised and performed well [14]. However, there are some risks in using single target features to detect targets or combining two features with equal weights if the features are not selected strictly. To ensure robust ship detection performance, especially in complex scenes, such as those with small islands and offshore floaters, it is necessary to efficiently combine multiple effective features together. Multiple features fusion is an effective approach to target detection. However, it is a great challenge to design the rule of fusing different polarimetric features. Focusing on the problem of ship detection from Pol-SAR images, a novel detection method based on the fusion of multiple channels data is presented in this paper.

The HH channel and extracted features—diplane scattering and helical factor—are used separately to detect target by a Cell Averaging (CA)-CFAR detector. Then a simple fusion process is applied to the detection results. Confirmed target pixels and potential target pixels (pixels which might be targets) are obtained after fusion. Then those potential target pixels will be further confirmed by comparing the difference degree. With comprehensive utilization of feature extraction, target detection, fusion of detection results and difference degree discrimination, this novel method is able to detect ships accurately. We also found that the method performs well in preserving the shapes of the target.

The paper is organized as follows: the polarimetric features extraction for ship detection is introduced in Section 2. The new fusion-based ship detection method including the difference degree aiming at Pol-SAR image is described in Section 3. In Section 4, the proposed ship detection method is verified and analyzed based on the measured data obtained in real applications. Some useful conclusions are drawn in Section 5.

## 2. Polarimetric Feature Extraction 

Polarimetric radar measures the complex scattering matrix of a medium with quad polarizations. The scattering matrix in the linear orthogonal polarization basis can be expressed as:

(1) $$S=\left[\begin{array}{cc} s_{hh} & s_{hv}\\ s_{vh} & s_{vv} \end{array}\right]$$

where *s_hv_* is the scattering element of horizontal transmitting and vertical receiving polarization, and the other three elements are defined similarly. In the case of reciprocal backscattering, *s_hv_* = *s_vh_*.

The main difference between ships and ocean is that the ships are of complex metal structures. They usually have three physical scattering models in Pol-SAR images: odd scattering, caused by the direct backscattering surface perpendicular to the radar beam; dihedral scattering, caused by the ship’s vertical conducting plates and the sea surface; and multiple scattering, caused by other structure on ship. Therefore, ships show larger coherent scattering than surrounding sea surface, where the wind-driven ocean waves are responsible for a smaller coherent scattering [15].

So far, based on the above differences, the polarimetric features for ship detection mainly include co-polarized phase difference, co-polarized ratio, co-polarized correlation coefficient, the proportion of different scattering mechanism and polarization entropy, *etc.* In addition to the above features, considering the difference between the ships and ocean, some of the typical polarimetric features for ship detection are introduced as follows.

### 2.1. Polarization Entropy

Polarization entropy is based on the coherency eigen values, a measurement to describe the statistical randomness of various scattering mechanisms. The coherency matrix ***T*** is shown and decomposed as:

(2) $$T=\sum_{i=1}^{3}\lambda_{i}\left(e_{i}\cdot e_{i}^{*T}\right)$$

where *λ_i_* is the eigenvalue of ***T***, and ***e**_i_* is the corresponding eigenvector. Therefore, the polarization entropy can be obtained by calculating the logarithmic sum of eigenvalues:

(3) $$H=-\sum_{i=1}^{3}p_{i}{\text{log}}_{3}p_{i},\;p_{i}=\lambda_{i}/\sum_{j=1}^{3}\lambda_{j}$$

The ocean is dominated by surface scattering with low entropy, while ships have complex scattering with accordingly high entropy. For low incidence angles up to 60°, ships are well discriminated by the polarization entropy method [5].

### 2.2. Co-Polarized Phase Difference

Co-polarized phase difference (CPD) [12] is the phase difference between co-polarized channels of HH and VV, which is defined as Equation (4). It has the value ranging from −*π* to *π*:

(4) $$\varphi_{\text{hh}\_\text{vv}}=angle\left(s_{hh}\right)-angle\left(s_{vv}\right)$$

where *angle*(⋅) means the phase of a complex number. CPD can be used to detect target ships, since CPD standard deviation values are high in areas where man-made metallic targets are present and low over the surrounding free sea surface [16]. The modulus of CPD also can be used for ship detection and it performs well.

### 2.3. Pauli Decomposition

The Pauli decomposition expresses the scattering matrix under so-called Pauli basis as follows:

(5) $$\left[S\right]=\left[\begin{array}{cc} s_{hh} & s_{hv}\\ s_{vh} & s_{vv} \end{array}\right]=\frac{a}{\sqrt{2}}\left[\begin{array}{cc} 1 & 0\\ 0 & 1 \end{array}\right]+\frac{b}{\sqrt{2}}\left[\begin{array}{cc} 1 & 0\\ 0 & -1 \end{array}\right]+\frac{c}{\sqrt{2}}\left[\begin{array}{cc} 0 & 1\\ 1 & 0 \end{array}\right]+\frac{d}{\sqrt{2}}\left[\begin{array}{cc} 0 & -j\\ j & 0 \end{array}\right]$$

where *a*, *b*, *c* and *d* are all complex numbers. Their values are respectively given by:

(6) $$a=\frac{s_{hh}+s_{vv}}{\sqrt{2}},\;b=\frac{s_{hh}-s_{vv}}{\sqrt{2}},\;c=\frac{s_{hv}+s_{vh}}{\sqrt{2}},\;d=\frac{s_{hv}-s_{vh}}{\sqrt{2}}$$

For reciprocal media, the four parameters can be simplified into three, that is *a*, *b* and *c*. They represent the coefficients of three categories of scattering components: surface scattering, dihedral scattering and the diplane scattering oriented at 45° [17]. The third component is denoted as cross-polarized, which is caused by volume scattering, complex structure targets and so on. Therefore diplane scattering of Pauli decomposition has favourable performance to detect ships. In Equation (5), |*a*|^2^, |*b*|^2^, |*c*|^2^ represent the contributions of three scattering components, respectively, which can be used as scattering mechanism expressions for target analysis.

### 2.4. Barnes-Holm Decomposition

Radar targets do not change according to the orientation, environment, radar frequency, waveforms, *etc.* Based on this rationale, Huynen proposed methods to extract physical characteristics and structural information for coherent and incoherent targets, respectively. Considering the existence of clutter, we use the incoherent decomposition. Huynen decomposition factorizes coherency scattering matrix ***T***_3_ into a rank 1 target ***T***_0_ which is pure scattering and a distributed roll-invariant target ***T**_N_*, the rank of which is larger than 1, so as to find a resolution to distinguish the wanted target from clutter environment. Based on the same structure, Barnes and Holm proposed a target decomposition theory which has preferably detection effect as well [17].

Average distributed targets are usually expressed by coherency matrix:

(7) $$T_{3}=\left[\begin{array}{ccc} 2\langle A_{0}\rangle & \langle C\rangle -j\langle D\rangle & \langle H\rangle +j\langle G\rangle \\ \langle C\rangle +j\langle D\rangle & \langle B_{0}\rangle +\langle B\rangle & \langle E\rangle +j\langle F\rangle \\ \langle H\rangle -j\langle G\rangle & \langle E\rangle -j\langle F\rangle & \langle B_{0}\rangle -\langle B\rangle \end{array}\right]=T_{0}+T_{N}$$

where 〈⋅〉 represents the ensemble average.

The structure proposed by Huynen is not unique. There are two other decomposition methods to make roll-invariant N-target, which are proposed by Barnes and Holm. One of them is adopted in this paper. One of the three normalized target vectors corresponding to ***T***_0_ is:

(8) $$\underline{k}=\frac{T_{3}\underline{q}}{\sqrt{{\underline{q}}^{T*}T_{3}\underline{q}}}=\frac{1}{\sqrt{2\left(\langle B_{0}\rangle -\langle F\rangle \right)}}\left[\begin{array}{c} \langle C\rangle -\langle G\rangle +j\langle H\rangle -j\langle D\rangle \\ \langle B_{0}\rangle +\langle B\rangle -\langle F\rangle +j\langle E\rangle \\ \langle E\rangle +j\langle B_{0}\rangle -j\langle B\rangle -j\langle F\rangle \end{array}\right]$$

where $\underline{q}$ belongs to the orthogonal space of the N-target, and it is given by:

(9) $$\underline{q}=\frac{1}{\sqrt{2}}\left[\begin{array}{c} 0\\ 1\\ j \end{array}\right]$$

This target vector is correspondent to non-symmetric components of target. The helical factor can be obtained from $\underline{k}$:

(10) $$T_{22}=\frac{{\left(\langle B_{0}\rangle +\langle B\rangle -\langle F\rangle \right)}^{2}+{\langle E\rangle }^{2}}{2\left(\langle B_{0}\rangle -\langle F\rangle \right)}$$

The purpose of Huynen and Barnes-Holm decomposition is to extract a scattering mechanism, which can be described by a single scattering matrix. It can be used for analysing man-made targets or areas. This type is usually characterised by a high density of identical targets, whereas natural scenes presents distributed scatterers which could be considered as a noise component by the two decompositions [17]. Therefore, it’s feasible to utilize Barnes-Holm decomposition to distinguish ships from ocean surface.

Almost all of the above polarimetric features can be used for distinguishing ships from the ocean clutter to a certain degree. At the same time, their performance varies under different scenes. To obtain robust detection results, several features should be coordinated applied. In this paper, the HH image, the diplane scattering coefficient and helical factor are utilized to detect ship in Pol-SAR images.

## 3. Fusion-Based Ship Detection Method

**Figure 1:**
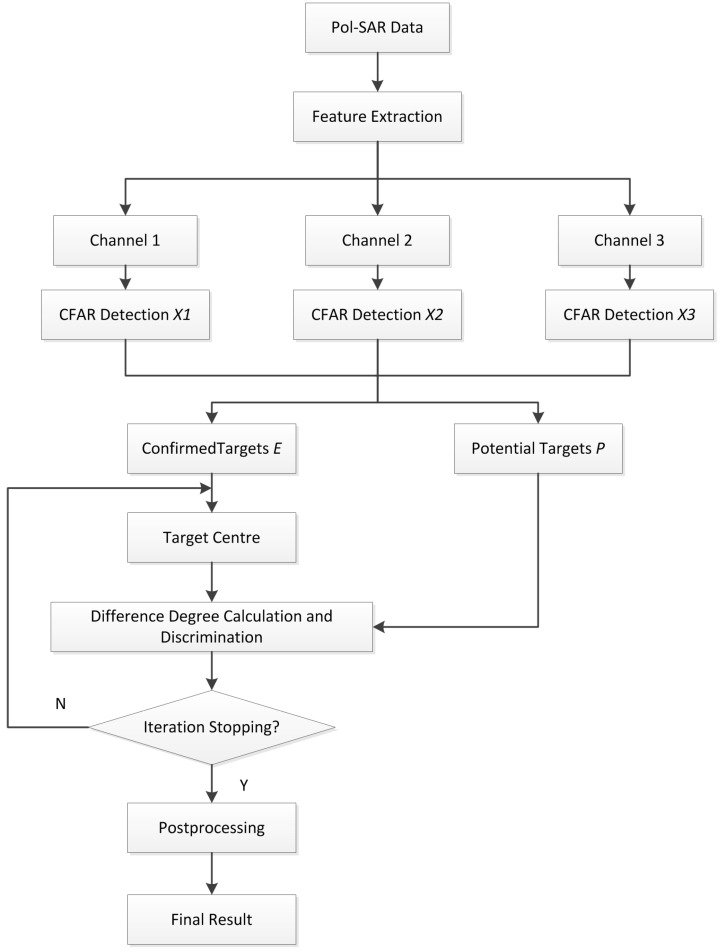
Flow chart of fusion-based detection.

A robust detector should not only find targets but also eliminate false alarms. For ship detection in complex scenes, it’s hard to eliminate the interferences from small islands and offshore floaters just using original images. An effective ship detector requires a combination of different features because each feature reveals only one-sided scattering characteristics. Since the differences between ships and ocean surface, small islands, floaters are multidimensional, more reliable detection results can be obtained by fusion processing. In this paper, the proposed detection method includes the process of polarimetric feature extraction, CFAR detection, fusion, potential targets recognition, *etc.* The novel fusion-based ship target detection method has the flow chart as shown in Figure 1.

### 3.1. Feature Extraction

Ships are different from the sea surface, offshore floaters and small islands in structure and material, which is the basis of ship detection from complex backgrounds. In order to demonstrate the differences between the complex structure of ships and those interferences, diplane scattering extracted by Pauli decomposition can be chosen as one of the detecting features. Helical factor provided by Barnes-Holm decomposition is related to helical type scattering, by which the man-made targets and ocean surface can be distinguished. This scattering also can be chosen to detect features [17]. In addition, echoes from different polarization channels reflect target characteristics and can also be treated as a kind of feature as well. Considering the influence of the surface capillary wave, the surface clutter from the VV channel is usually higher than that from HH channel, which causes the ratio of signal to clutter (SCR) in VV channel lower than HH channel. Therefore, HH channel, diplane scattering of Pauli decomposition and helical factor of Barnes-Holm decomposition are adopted to detect ships.

### 3.2. CFAR Detection

The information of both ships and other man-made targets lie in the three dimensional feature space of HH, diplane scattering and helical factor. The CFAR detector is employed in this paper. According to different application scenes, CFAR detector has different implementations, such as Greatest Of selection CFAR (GO-CFAR), Smallest Of selection CFAR (SO-CFAR), Order Statistic CFAR (OS-CFAR) and Knowledge Based CFAR (KB-CFAR). Each of them can be used in particular scenes, especially in complex inhomogeneous backgrounds. Considering that SAR image of ocean scene has homogeneous scattering characteristics, a simple CA-CFAR can be used for ship detection from each channel in this method.

Assume the probability density function (PDF) of background is *p*(*x*). Background and target can be separated by threshold *t*, and then the probability of false alarm is:

(11) $$P_{fa}=\int_{t}^{\infty }p\left(x\right)dx$$

**Figure 2:**
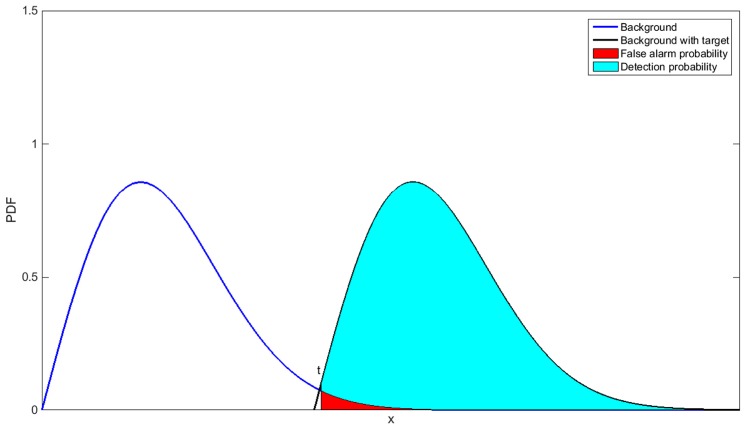
Expression of detection probability and false alarm probability.

The detection threshold *t* can be deduced by the above formula. Figure 2 shows the expression of detection probability and false alarm probability.

CFAR detection based on Gaussian distribution is used in HH channel and feature channels. In case of helical factor channel, since it’s hard to know the distribution underlying the clutter, the result of CA-CFAR is only an approximation. After the CFAR detection, morphological filtering is employed to obtain better detection performance.

### 3.3. Fusion Processing

After CFAR processing, some suspected areas can be obtained. For the purpose of comprehensively utilizing detection results from different feature channels, fusion processing is applied. The detection results can be classified into target pixels, background pixels and potential target pixels, among which confirmed target pixels are those judged as target in every feature channel. The set of confirmed target pixels is obtained by Equation (12):

(12) $$E=\left\{e_{i}|e_{i}=x_{1i}\cap x_{2i}\cap x_{3i},x_{1i}\in X_{1},x_{2i}\in X_{2},x_{3i}\in X_{3}\right\}$$

where *E* is the set of confirmed target pixels, and *X*_1_, *X*_2_, *X*_3_ denote CFAR detection results from different channels of HH, diplane scattering and helical factor, respectively. We also have *X*_1_ = {*x*_1*i*_}, *X*_2_ = {*x*_2*i*_}, *X*_3_ = {*x*_3*i*_}. Confirmed target pixels will be used in discriminating target pixels from potential target pixels.

Potential target pixels are those judged as target by one or two channels, which need further confirmation. The set of potential target pixels can be expressed as follows:

(13) $$P=\left\{p_{i}|p_{i}=\left(x_{1i}-e_{i}\right)\cup \left(x_{2i}-e_{i}\right)\cup \left(x_{3i}-e_{i}\right),x_{1i}\in X_{1},x_{2i}\in X_{2},x_{3i}\in X_{3},e_{i}\in E\right\}$$

where *P* is the set of potential target pixels, and *e_i_* is an element of the set *E*, namely the confirmed target pixels.

Except for confirmed target pixels and potential target pixels, others are background pixels. By using fusion processing, three detection results are combined into one result, which contains confirmed targets, background, and potential target pixels.

### 3.4. Potential Target Pixels Judgment

Each type of target has its own scattering characteristic. To describe the difference between targets quantitatively, a description of difference degree is employed [18]. Suppose 〈*T_i_*〉 and 〈*T_j_*〉 are coherency matrices of targets, whose total powers are *P_i_* and *P_j_* respectively. The difference degree *d_ij_* is defined as follows:

(14) dij=(1−|〈Ti〉‖〈Ti〉‖F⋅〈Tj*〉‖〈Tj〉‖F|)+(1−2(PiPj+PjPi))

where (⋅) is the inner product operator, and ‖⋅‖_*F*_ is Frobenius norm. The total power *P*, also known as *span*, is defined by Equation (15):

(15) $$span={\left|s_{hh}\right|}^{2}+{\left|s_{vv}\right|}^{2}+2{\left|s_{hv}\right|}^{2}$$

Equation (15) can be separated into two parts: the former part $\left(1-\left|\left(\langle T_{i}\rangle /{\left\|\langle T_{i}\rangle \right\|}_{F}\right)\cdot \left(\langle T_{j}^{*}\rangle /{\left\|\langle T_{j}\rangle \right\|}_{F}\right)\right|\right)$ shows correlation between two coherency matrices while the latter $\left(1-2/\left(P_{i}/P_{i}+P_{j}/P_{i}\right)\right)$ reflects echo power difference. The difference degree has the clear range of 0–2. When 〈*T_i_*〉 = 〈*T_j_*〉 is satisfied, the distance *d_ij_* will be zero.

Due to the huge difference between ships and backgrounds in structure and material, the ships’ echoes are different from those of the sea surface and other non-ship targets, such as islands and floaters *etc.*, which makes it feasible to distinguish ship pixels from potential target pixels. In this paper, an unsupervised iterative classification based on difference degree is applied to discriminate potential target pixels.

The confirmed elements in E, as samples of ships, are used to calculate the average class-centre of ships. Then the difference degrees between ship class and potential target pixels in the set *P* are obtained. Comparing the difference degrees with discrimination threshold, the potential target pixels can be classified into set *E* and labeled as targets if their difference degrees are under the threshold. After the judgment, the class-centre of ships should be recalculated. Generally a stable result can be obtained after 3–4 iterations.

## 4. Experimental Verification

**Figure 3:**
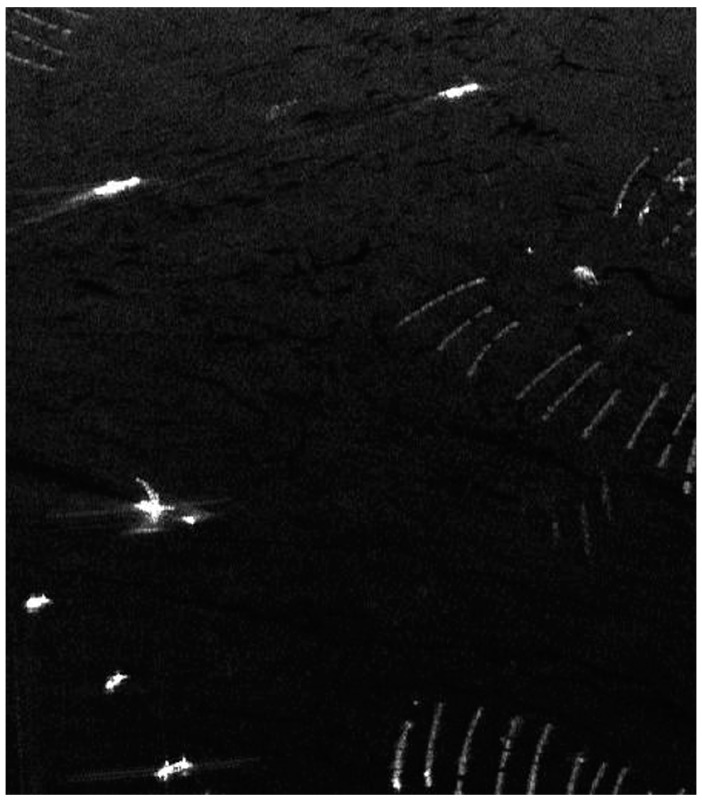
HH channel image.

To validate and test the performance of the proposed ship detection method, the NASA/JPL AIRSAR L-band data of Etajima, near Hiroshima Bay, was used, which was acquired in 2000. There were seven ships and some other interferences including a small island and some floaters in the scene. The HH channel is shown in Figure 3.

The experimental verification included two aspects, which were ship detection performance and target shape preserving.

### 4.1. Ship Detection Performance

To validate the ship detection performance, the comparison between the fusion-based method and two other methods—K-means clustering and Notch Filter—is made in this paper.

#### 4.1.1. Fusion-Based Ship Detection

**Figure 4:**
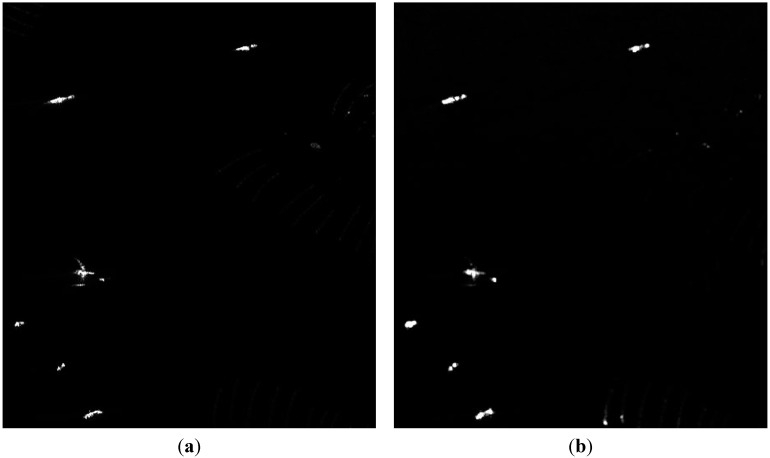
Extracted features: (**a**) The diplane scattering; (**b**) Helical factor.

According to the procedure shown in Section 3, the first step was to extract polarimetric features. The diplane scattering and helical factor were extracted, which are shown in Figure 4.

**Figure 5:**
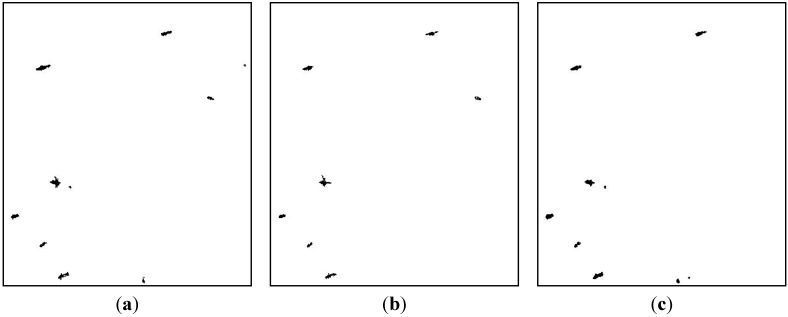
CFAR detection result: (**a**) HH channel; (**b**) Diplane scattering channel; (**c**) Helical factor channel.

It can be seen in Figure 4 that the ships have higher diplane scattering and helical factor than the ocean surface, the small island and other man-made floaters. The extracted features will be available for ship detection. At the same time, the island and some floaters in Figure 4 have higher diplane scattering and helical factor than ocean surface, which could cause false alarms. After that, CFAR detector is applied for HH channel, diplane scattering channel, and helical factor channel respectively. The detection results are shown in Figure 5.

In Figure 5a, seven ships are detected approximately. Meanwhile, the small island and floaters on the top right and bottom are detected. In Figure 5b, the island is detected, whereas a small ship is missing. In Figure 5c seven ships are all detected, with some floaters on the bottom. Although the main targets are detected by the three feature channels, there are great differences in ship shapes. Fusion processing is necessary for the further application such as target recognition or identification.

**Figure 6:**
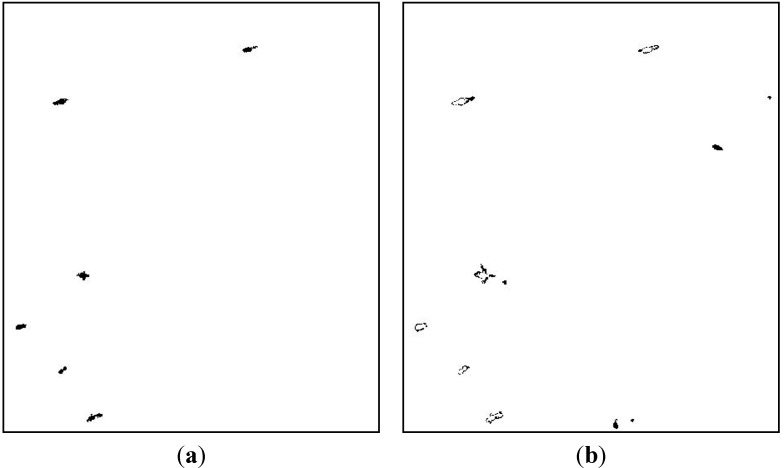
Result of fusion processing: (**a**) Confirmed target pixels; (**b**) Potential target pixels.

Based on the detection results of three channels, confirmed target pixels and potential target pixels are acquired by Equations (11) and (12), as shown in Figure 6a,b. Pixels in Figure 6b contain targets and false alarms. As can be seen, many pixels around confirmed targets are divided into potential target class. Further judgment of potential target pixels can detect both the missed targets in Figure 6a and the missed pixels around confirmed targets, which will be useful to maintain target shape.

**Figure 7:**
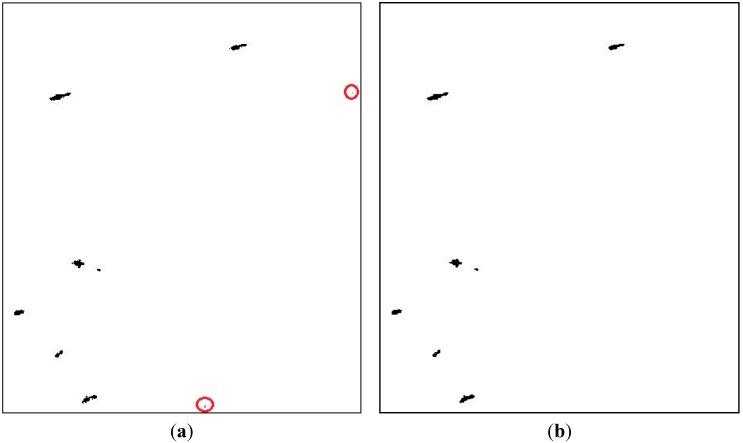
Ship detection results: (**a**) After iterative modification; (**b**) After morphological filtering.

Then, the difference degrees between potential target pixels and class-centre of confirmed targets will be calculated and applied to iterative modification. If the difference degree is less than a threshold, the potential target pixel will be classified into the target class. Then the target centre will be modified. Generally, 3–4 rounds of iteration are enough to guarantee convergence. After iterative modification, the detection result is shown as Figure 7a, in which the missing small dim target is detected and other potential pixels are classified as background. Finally, a morphological filter is used to remove the isolated pixels and very small blocks. It should be noted that though detection masks with the other algorithms do not require morphological filter, due to the complexity of fusion-based method, this algorithm relies on it to get better detection performance and maintain the integrity of ship. The minimum size of vessels that can be detected with this algorithm is 10 pixels, *i.e.*, the minimum area of ships.The final detection result is shown in Figure 7b. It can be shown that all seven ships are detected and those two remaining false alarm are filtered out.

#### 4.1.2. Ship Detection Based on K-means Clustering

**Figure 8:**
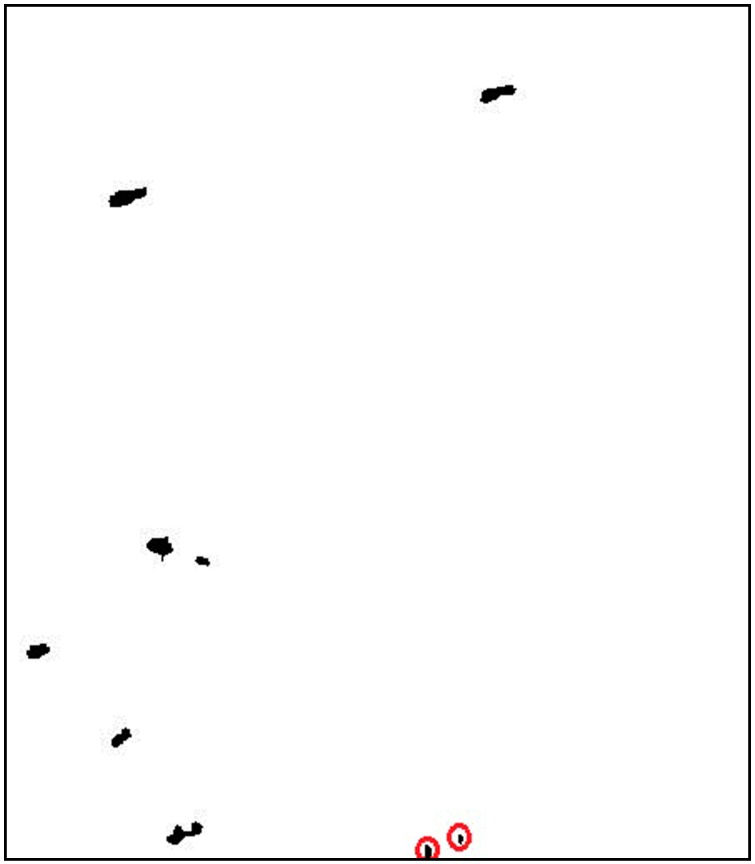
Detection result of K-means clustering.

Lu [19] conducted ship detection based on the K-means clustering, which also obtained good detection results by using the selected classification features of dihedral scattering and co-polarized phase difference. The detection result is shown in Figure 8. From Figure 8, it can be seen that the seven ships are all detected, but at the same time there are two false alarms, which are circled out in Figure 8.

#### 4.1.3. Ship Detection Based on Notch Filter

The general idea of ship detection based on the Notch Filter is to create a filter, reject the sea return and extract the remaining features, not only ships but also floaters or any other structures located on the sea [13]. Ships and other objects can be regarded as the randomly distributed targets in the marine environment. A feature partial scattering vector is introduced as:

(16) $$\underline{t}=Trace(\left[C\right]\Psi )$$

where *C* is covariance matrix, Ψ is a complete set of 3 × 3 basis matrices under a Hermitian inner product, shown as:

(17) $$\Psi =\left\{\left[\begin{array}{ccc} 1 & 0 & 0\\ 0 & 0 & 0\\ 0 & 0 & 0 \end{array}\right],\left[\begin{array}{ccc} 0 & 0 & 0\\ 0 & 1 & 0\\ 0 & 0 & 0 \end{array}\right],\left[\begin{array}{ccc} 0 & 0 & 0\\ 0 & 0 & 0\\ 0 & 0 & 1 \end{array}\right],\left[\begin{array}{ccc} 0 & 0 & 0\\ 1 & 0 & 0\\ 0 & 0 & 0 \end{array}\right],\left[\begin{array}{ccc} 0 & 0 & 0\\ 0 & 0 & 0\\ 1 & 0 & 0 \end{array}\right],\left[\begin{array}{ccc} 0 & 0 & 0\\ 0 & 0 & 1\\ 0 & 0 & 0 \end{array}\right]\right\}$$

The partial scattering vector of the sea clutter can be completely described by a vector in six dimensional complex space ${\underline{\hat{t}}}_{\;\;sea}\in {\mathbb{C}}^{6}$.

The Notch Filter detector can be denoted as:

(18) $$\gamma =\frac{1}{\sqrt{1+RedR\cdot \frac{P_{sea}}{P_{T}}}}>T$$

where *RedR* stands for Reduction Ratio, $P_{sea}={\left|{\underline{t}}^{*}{\underline{\hat{t}}}_{\;\;sea}\right|}^{2}$ is the sea clutter power, and $P_{T}={\underline{t}}^{*}\underline{t}-{\left|{\underline{t}}^{*}{\underline{\hat{t}}}_{\;\;sea}\right|}^{2}$ is the power of the “non-sea” targets. Considering the GP-PNF (Geometrical Perturbation-Polarimetric Notch Filter) has two independent parameters, the threshold *T* can be chosen arbitrarily (e.g., *T* = 0.98). The *RedR* is set locally. Based on the minimum target of interest $P_{T}^{\text{min}}$, considering the expected backscattering of vessels, *RedR* can be obtained by Equation (19):

(19) $$RedR=P_{T}^{\text{min}}\left(\frac{1}{T^{2}}-1\right)$$

The detection result is shown as follows:

**Figure 9:**
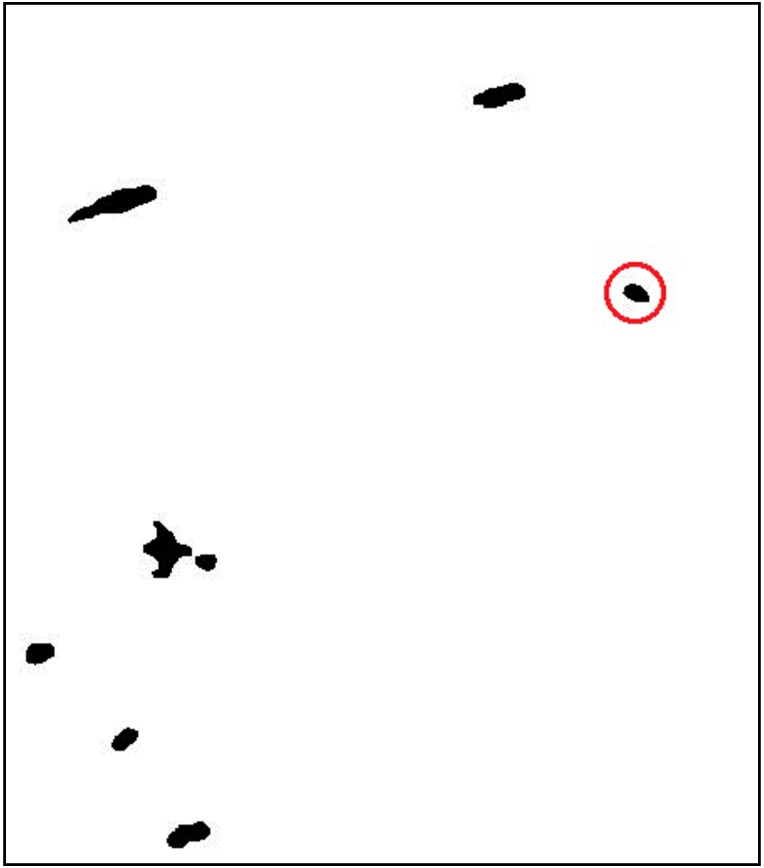
Detection result of Notch Filter.

It can be seen from Figure 9, seven ships are detected in addition to a small island. The effect of this method is significant for detecting non-sea targets. However it is not accurate enough to distinguish ships from interferences on the sea. Seven ship targets can be detected by all three methods, however the shapes of the targets deviate a lot from each other. In order to evaluate the three detection methods further, the performance metric of shape preserving ability is proposed.

### 4.2. Target Shape Preserving

After target detection, the result may be used for target recognition in the subsequent processing. The shape of target ships may influence the performance of successive processing. The detection result is compared to ships models to evaluate the shape preserving ability.

**Figure 10:**
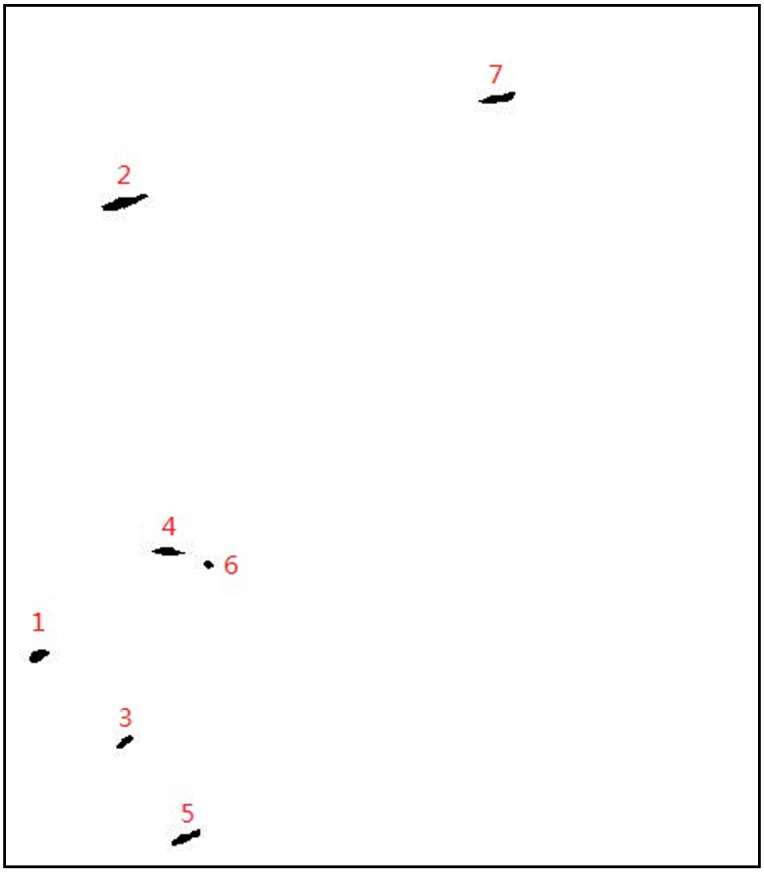
Ships models.

First, the ship models are achieved manually in our test. They were selected visually by comparing several different polarimetric features of this Pol-SAR image. Ship models were labelled as number 1 to 7 in Figure 10.

Then the target preserving performance of the three methods is compared by counting the correctly detected pixels, the missed pixels and the false pixels, respectively. The counting result is shown in Table 1. In this table, *N_model_* is the pixel number of the ship models. *N_det_* is the pixel number of the detected ship 1 to 7. *N_cor_* is the number of correct pixels, which are the intersection of ship model and each detected ship. *N_fal_* = *N_det_* − *N_cor_* is the number of false alarm pixels. *N_miss_* = *N_model_* − *N_cor_* is the number of missed pixels.

**Table 1:** Pixels counting of detected ships numbered as Ships 1–7.

Ship No.	*N_model_*	K-Means Clustering				The Notch Filter				Fusion-Based Method			
		*N_det_*	*N_cor_*	*N_fal_*	*N_miss_*	*N_det_*	*N_cor_*	*N_fal_*	*N_miss_*	*N_det_*	*N_cor_*	*N_fal_*	*N_miss_*
1	64	93	62	29	2	176	64	112	0	70	57	6	7
2	128	191	102	63	26	518	128	390	0	136	116	8	12
3	40	75	40	35	0	144	40	104	0	50	35	10	5
4	65	140	56	75	9	473	65	408	0	101	53	36	12
5	75	145	65	70	10	266	75	191	0	99	71	24	4
6	21	38	18	17	3	102	21	81	0	22	10	1	11
7	85	114	69	29	16	304	85	219	0	103	66	18	19

**Figure 11:**
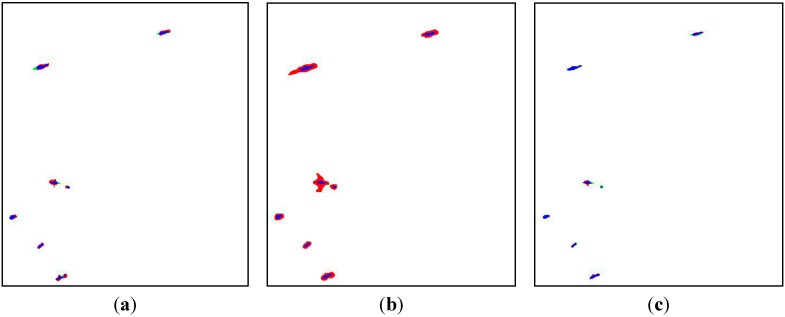
Shape preserving mappings: (**a**) K-means clustering; (**b**) The Notch Filter; (**c**) Fusion-based method.

The effect of ship shape preservation is expressed by color mapping in Figure 11, in which the correctly detected pixels are marked blue, the missed pixels are marked green and the false alarm pixels are marked red. It is clear that the detected ships of novel fusion-based method are closer to the models.

Based on the data in Table 1, a quality factor *P_fom_* is calculated as a metric to measure the shape preserving performance. *P_fom_* is defined as:

(20) $$P_{fom}=\frac{N_{cor}}{N_{false}+N_{miss}+N_{cor}}$$

Obviously, a higher value of *P_fom_* represents a better shape preserving ability. Quality factors of the seven detected ships are shown in Table 2.

**Table 2:** Shape preserving quality factors of detected ships numbered 1–7.

Ship No.	K-Means Clustering	The Notch Filter	Fusion-Based Method
1	66.7	36.4	81.4
2	53.4	24.7	85.3
3	53.3	27.8	70.0
4	40.0	13.7	52.5
5	44.8	28.2	71.7
6	47.4	20.6	45.5
7	60.5	28.0	64.1
**Average**	51.8	24.1	70.2

The total average *P_fom_* of K-means clustering, the Notch Filter and fusion-based method are 51.8%, 24.1% and 70.2%, respectively. Therefore, the fusion-based method has the potential to preserve the ship shapes with highest accuracy because of the introduction of iterative modification.

## 5. Conclusions

In order to face the challenge of ship detection in complex scenes, a novel fusion-based ship detection method for Pol-SAR images is proposed in this paper. This method includes the processing of CFAR detection, results fusion and morphological filtering. Different detection results can be obtained by using CFAR detector and morphological filtering on three dimensional feature spaces of HH, diplane scattering and helical factor. Based on the different results, the fusion process obtains the confirmed targets and other potential pixels. After the discrimination of potential pixels based on difference degree and morphological filtering, the final result is obtained. The application to processing measured AIRSAR data shows that all seven ships are detected without any false alarm. For comparison, The K-means clustering method and the Notch Filter method are also tested. However, one or two false alarms result at the same time. The experimental results with one AIRSAR acquisition indicate that the novel method we propose has better performance in ship detection and recognition in complex scenes. Furthermore, the shape preserving ability is enhanced with a quality factor of 70.2%, which is 18.4% higher than that of K-means clustering method, and 46.1% higher than that of the Notch Filter method. Experimental result shows that the fusion-based method can effectively reduce the false alarms, without distorting ship shapes.

## References

[1] Copeland A.C., Ravichan G., Trivedi M.M. (1995). Localized radon transform-based detection of ship wakes in SAR Images. IEEE Trans. Geosci. Remote Sens..

[2] Vachon P.W., Campbell J., Bjerklund C. (1997). Ship detection by the RADARSAT SAR: Validation of detection model predictions. Can. J. Remote Sens..

[3] Liu C., Vachon P.W., Geling G.W. (2005). Improved ship detection with airborne polarimetric SAR data. Can. J. Remote Sens..

[4] Touzi R., Charbonneau F., Hawkins R.K., Murnaghan K., Kavoun X. Ship-sea contrast optimization when using polarimetric SARs. Proceedings of the IGARSS’ 01.

[5] Ringrose R., Harris N. Ship detection using polarimetric SAR data. Proceedings of the CEOS SAR workshop.

[6] Cameron W.L., Youssef N.N., Leung L.K. (1996). Simulated polarimetric signatures of primitive geometrical shapes. IEEE Trans. Geosci. Remote Sens..

[7] Touzi R. (2002). Characterization of target symmetric scattering using polarimetric SARs. IEEE Trans. Geosci. Remote Sens..

[8] Nunziata F., Migliaccio M., Brown C.E. (2012). Reflection symmetry for polarimetric observation of man-made metallic targets at sea. IEEE J. Ocean. Eng..

[9] Nunziata F., Migliaccio M. (2013). On the COSMO-SkyMed PingPong Mode to observe metallic targets at sea. IEEE J. Ocean. Eng..

[10] Yeremy M., Campbell J.W.M., Mattar K., Potter T. (2001). Ocean surveillance with polarimetric SAR. Can. J. Remote Sens..

[11] Li H.Y., He Y.J., Wang W.G. (2009). Improving ship detection with polarimetric SAR based on 2-D convolution between co-polarization channels. Sensors.

[12] Wang W.G., Wang J., Lei P., Mao S.Y. (2008). A new ship detection method based on polarimetric SAR classification. Chin. J. Electron..

[13] Marino A. (2013). A notch filter for ship detection with polarimetric SAR data. IEEE J. Sel. Top. Appl. Earth Obs. Remote Sens..

[14] Marino A., Hajnsek I. (2015). Statistical tests for a ship detector based on the Polarimetric Notch Filter. IEEE Trans. Geosci. Remote Sens..

[15] Velotto D., Soccorsi M., Lehner S. (2014). Azimuth ambiguities removal for ship detection using full polarimetric X-Band SAR data. IEEE Trans. Geosci. Remote Sens..

[16] Migliaccio M., Nunziata F., Montuori A., Li X., Pichel W.G. (2011). A multifrequency polarimetric SAR processing chain to observe oil fields in the Gulf of Mexico. IEEE Trans. Geosci. Remote Sens..

[17] Polarimetry Tutorial Part 1-Tutorial on Radar Polarimetry: Polarimetric Decomposition. https://earth.esa.int/web/polsarpro/polarimetry-tutorial.

[18] Wang W.G., Wang J., Mao S.Y. Classification of polarimetric SAR images based on difference degree. Proceedings of the EUSAR.

[19] Lu F., Wang W.G., Xing K.Q., Lin X.X. The ship discrimination based on D-S evidence theory. Proceedings of the 11th International Conference on Signal Processing.

